# Pre- and Posttreatment With Edaravone Protects CA1 Hippocampus and Enhances Neurogenesis in the Subgranular Zone of Dentate Gyrus After Transient Global Cerebral Ischemia in Rats

**DOI:** 10.1177/1759091414558417

**Published:** 2014-11-10

**Authors:** Shan Lei, Pengbo Zhang, Weisong Li, Ming Gao, Xijing He, Juan Zheng, Xu Li, Xiao Wang, Ning Wang, Junfeng Zhang, Cunfang Qi, Haixia Lu, Xinlin Chen, Yong Liu

**Affiliations:** 1 Department of Anesthesiology, Second Affiliated Hospital of Xi’an Jiaotong University School of Medicine, Xi’an, China; 2 Institute of Neurobiology, National Key Academic Subject of Physiology of Xi’an Jiaotong University School of Medicine, Xi’an, China; 3 Department of Orthopedics, Second Affiliated Hospital of Xi’an Jiaotong University School of Medicine, Xi’an, China; 4 Department of Anatomy, Xi’an Medical University, Xi'an, China

**Keywords:** neurogenesis, cerebral ischemia, neural stem/progenitor cells, neuroprotection, edaravone, reactive oxygen species

## Abstract

Edaravone is clinically used for treatment of patients with acute cerebral infarction. However, the effect of double application of edaravone on neurogenesis in the hippocampus following ischemia remains unknown. In the present study, we explored whether pre- and posttreatment of edaravone had any effect on neural stem/progenitor cells (NSPCs) in the subgranular zone of hippocampus in a rat model of transient global cerebral ischemia and elucidated the potential mechanism of its effects. Male Sprague-Dawley rats were divided into three groups: sham-operated (*n* = 15), control (*n* = 15), and edaravone-treated (*n* = 15) groups. Newly generated cells were labeled by 5-bromo-2-deoxyuridine. Immunohistochemistry was used to detect neurogenesis. Terminal deoxynucleotidyl transferase-mediated dUTP-biotin nick-end labeling was used to detect cell apoptosis. Reactive oxygen species (ROS) were detected by 2,7-dichlorofluorescien diacetate assay in NSPCs *in vitro*. Hypoxia-inducible factor-1α (HIF-1α) and cleaved caspase-3 proteins were quantified by western blot analysis. Treatment with edaravone significantly increased the number of NSPCs and newly generated neurons in the subgranular zone (*p* < .05). Treatment with edaravone also decreased apoptosis of NSPCs (*p* < .01). Furthermore, treatment with edaravone significantly decreased ROS generation and inhibited HIF-1α and cleaved caspase-3 protein expressions. These findings indicate that pre- and posttreatment with edaravone enhances neurogenesis by protecting NSPCs from apoptosis in the hippocampus, which is probably mediated by decreasing ROS generation and inhibiting protein expressions of HIF-1α and cleaved caspase-3 after cerebral ischemia.

## Introduction

Global cerebral ischemia is a clinical outcome of cardiac arrest, severe hypotension, or certain operations, such as cardiopulmonary bypass and cerebral intervention, which deprive the brain of oxygen and glucose. It causes severe damage to pyramidal neurons of the CA1 region and usually results in residual neurological deficits following recovery from ischemia (Pulsinelli and Brierley, 1979; Nitatori et al., 1995). The subgranular zone (SGZ) of the dentate gyrus (DG) is the most important regenerative center in the hippocampus. Neural stem/progenitor cells (NSPCs) derived from the SGZ migrate into the DG of the hippocampus, where some of them mature into granule neurons. This neurogenesis process is important for learning and synaptic plasticity under physiological conditions (Iscru et al., 2013). Increased neurogenesis originating from NSPCs in the SGZ of the adult brain has been observed in a variety of mammals and humans following cerebral ischemia (Eriksson et al., 1998; Gage, 2000). The capacity of the adult brain to induce spontaneous neurogenesis is closely associated with recovery after ischemia (Liu et al., 1998; Nakatomi et al., 2002; Sharp et al., 2002; Alvarez-Buylla and Lim, 2004; Di Filippo et al., 2008; Zhang et al., 2008). Thus, neuroprotective and neurorestorative medication for treatment after cerebral ischemia should be developed.

Apoptosis is an essential process that occurs during normal development to maintain homeostasis and occurs under pathological conditions (Guan et al., 2009). After global cerebral ischemia, the larger part of the newly generated NSPCs in the hippocampus dies through apoptosis within weeks (Bingham et al., 2005; Sun et al., 2012). Caspases are a family of cysteine proteinases that play a central role in the control of apoptosis (Springer et al., 2001; Jin et al., 2003). Activation of caspase-3 results in the degradation of a number of intracellular and cytoskeletal protein substrates as part of an ordered process of cellular disassembly, leading to apoptotic cell death (Mancini et al., 1998; Sun et al., 1999). In addition, caspase-3 mediates the loss of neurons in the CA1 region and neurogenic cells in the SGZ (Bingham et al., 2005), and there is a causal relationship between hypoxia-inducible factor-1α (HIF-1α) and caspase-3 induction via HIF-1α functional binding to the caspase-3 gene promoter (Van Hoecke et al., 2007). Moreover, accumulating evidence shows the involvement of reactive oxygen species (ROS) in the activation of signaling components upstream of HIF-1α, such as hydroxylases and kinases (Hwang et al., 2008; Koshikawa et al., 2009).

Edaravone (3-methyl-1-phenyl-2-pyrazolin-5-one) is a free radical scavenger that can cross the blood–brain barrier and interact with a variety of free radicals to reduce ROS generation (Aoyama et al., 2008). Edaravone has a neuroprotective role in focal cerebral ischemia in animals and in patients suffering from acute cerebral infarction (Kawai et al., 1997; Amemiya et al., 2005; Watanabe et al., 2008). However, the effect of pre- and posttreatment with edaravone on neurogenesis in the hippocampus after transient global cerebral ischemia is unknown. In the present study, we investigated whether edaravone administration had any effect on NSPCs in the SGZ after transient global cerebral ischemia and explored whether intracellular ROS and the HIF-1 signaling pathway were involved in the mechanism of action of edaravone.

## Materials and Methods

### Global Cerebral Ischemia Model

The Experimental Animal Center of Xi’an Jiaotong University School of Medicine (Certificate No. 22-9601018) provided all the animals. All studies were conducted in accordance with the National Institutes of Health Guide for the Care and Use of Laboratory Animals (NIH Publications No. 80-23). The Animal Care and Use Committee of Xi’an Jiaotong University School of Medicine approved the experimental protocols. Adult male Sprague-Dawley rats, weighing 200–250 g, were maintained on a 12-hr light/dark cycle with free access to food and water (Zheng et al., 2012). Transient brain ischemia (10 min) was induced by the four-vessel occlusion method, as described previously by Pulsinelli and Brierley (1979) and Yan et al. (2007). Briefly, rats were anesthetized with pentobarbital sodium (40 mg kg^−1^, intraperitoneally). The vertebral arteries were irreversibly electrocauterized. The common carotid arteries were exposed, and a small-diameter silk thread looped around each artery to facilitate subsequent occlusion. Rats were allowed to recover for 24 hr. Transient global cerebral ischemia was induced by simultaneously clamping the bilateral common carotid arteries for 10 min with two microvascular clamps. Rats whose pupils were dilated and unresponsive to light and that showed increased respiration were selected for the experiment. Sham-operated rats received the surgical procedures except that the vertebral arteries were not electrocauterized and the common carotid arteries were not clamped. Rats in the edaravone group (*n* = 15) received an intraperitoneal injection of edaravone (3.0 mg kg^−1^; Boda, Jilin, China) 30 min before clamping the common carotid arteries and 30 min after reperfusion. This treatment schedule and dosage were based on the pharmacokinetic profile of edaravone supplied by the manufacturer and previous studies (Shichinohe et al., 2004; Nakajima et al., 2005; Akiyama and Miwa, 2007; Lu et al., 2012). Rats in the control group (operated on, no edaravone treatment; *n* = 15) or sham-operated group (*n* = 15) received intraperitoneal injection of normal saline using the same volume as the edaravone group. During ischemia and reperfusion, the rectal temperature was maintained at 37 ± 0.5℃. 5-bromo-2-deoxyuridine (BrdU, 50 mg kg^−1^; Sigma-Aldrich, USA) was injected intraperitoneally at the onset of global cerebral ischemia and then once daily for 7 or 14 consecutive days (Zhang et al., 2001). The rats were sacrificed at 7, 14, and 21 days after ischemia by excessive anesthesia, 2 hr after the final BrdU injection. Neurological score, which is a composite of motor (muscle status and abnormal movement), sensory (visual, tactile, and proprioceptive), reflex, and balance tests, was assessed by a blinded investigator according to the report by Beck et al. (2007) at 1, 3, 7, 14, and 21 days after ischemia. A maximum score of 21 is considered neurologically normal, whereas a score of 0 is considered as brain dead.

### Tissue Preparations and Histological Assessment

Seven, fourteen, and twenty-one days after clamping the bilateral common carotid arteries, rats of each group (*n* = 5 per time point) were killed by excessive anesthesia with pentobarbital sodium (40 mg/kg, intraperitoneally) and perfused transcardially with normal saline followed by 4% paraformaldehyde in phosphate-buffered saline (PBS). The brains were postfixed with 4% paraformaldehyde for 24 hr and embedded in paraffin. Finally, each sample from bregma −2.0 mm to bregma −4.0 mm was cut into 7 -µm-thick consecutive coronal sections using a microtome (HM340, Microm, Germany). For histological assessment, cresyl violet was used to stain the sections, which were observed under an optical microscope at 7, 14, and 21 days after ischemia. The length of the damaged CA1 pyramidal cell layer was measured under × 400 magnification and expressed as the percentage of the entire CA1 subregion. The severity of neuronal damage was graded as grade 0, *no damage to any hippocampal subregion*; grade 1, *scattered ischemic neurons in the CA1 subregion*; grade 2, *moderate ischemic damage*; grade 3, *whole pyramidal cell damage in the CA1 subregion*; and grade 4, *extensive cell damage in all hippocampal regions* (Sugawara et al., 2000; Lee et al., 2009).

### Immunohistochemistry and Terminal dUTP Nick-End Labeling Staining

The tissue sections were subjected to microwave heat-induced epitope retrieval. After blocking with 4% goat serum in PBS containing 0.3% Triton X-100 for 1 hr, the sections were incubated with primary antibodies at 4℃ overnight. The primary antibodies included rat polyclonal anti-BrdU antibody (1:1,000; Abcam, UK), rabbit anti-doublecortin (DCX) polyclonal antibody (1:1,000; Abcam, UK), mouse monoclonal anti-neuron-specific nuclear protein (NeuN) antibody (1:1,000; Chemicon, USA), and mouse monoclonal anti-glial fibrillary acidic protein (GFAP) antibody (1:800; Sigma, St. Louis, MO, USA). The sections were then washed with PBS and incubated with secondary antibodies [fluorescein isothiocyanate (FITC) or tetramethylrhodamine isothiocyanate (TRITC)-conjugated IgG, or biotinylated IgG] for 2 hr at room temperature. To detect apoptosis of newly generated cells in the SGZ, the BrdU-stained sections were labeled using the terminal dUTP nick-end labeling (TUNEL) assay, according to the instruction of in situ Cell Death Detection Kit (Roche, Germany), and negative/positive controls were also used according to the instructions. A laser confocal microscope (TSC SP2, Leica, Manheim, Germany) detected the fluorescence signals.

### NSPC Culture and Hypoxic Exposure

NSPCs were isolated from the cortex of Kunming mice on gestation Day 14 and cultured in T25 flasks, as described previously (Chen et al., 2010). After 5–7 days, the primary neurospheres were mechanically dissociated and triturated. Single cells were resuspended at a density of 1 × 10^5^ cells/mL and cultured for 3 to 4 days (passage 1 neurospheres). Passage 1 cells at a density of 5,000 cells/mL in serum-free medium were cultured in 96-well plates for 3 days, and then incubated with edaravone (0 μM or 100 μM, Boda, Jilin, China) under hypoxia (0.3% O_2_/94.7% N_2_/5% CO_2_) or without edaravone under normoxia (5% CO_2_/95% air) for 24 hr, as reported previously (Chen et al., 2010; Tian et al., 2010; Yoneyama et al., 2010; Zhao et al., 2012). An oxygen meter (CY-100B, Hangzhou Lihua Sci-technology Company, Hangzhou, China) monitored the percentage of oxygen in the chamber continuously. The cells were dissociated using 0.05% trypsin and 200 μM EDTA for 10 min at 37℃, followed by 75% ice-cold ethanol fixation overnight at −20℃. Immunocytochemical analysis was used to characterize the cultured cells. The primary antibodies included mouse monoclonal anti-nestin antibody (1:500; Chemicon, Temecula, CA, USA), rabbit polyclonal anti-HIF-1α antibody (1:1,000; Abcam, Cambridge, UK), mouse monoclonal anti-β-tubulin III antibody (1:1000; Sigma, St. Louis, MO, USA), and mouse monoclonal anti-GFAP antibody (1:800; Sigma, St. Louis, MO, USA). The nuclei were counterstained with propidium iodide (PI, 50 mg/mL; Sigma, St Louis, MO, USA) or DAPI (Sigma, St Louis, MO, USA). At least three independent experiments were performed for each assay.

### Measurement of ROS in NSPCs

The dye 2,7-dichlorofluorescien diacetate (DCF-DA; Sigma-Aldrich, USA), which is intracellularly oxidized to the fluorescent 2,7-dichlorofluorescien (DCF) in the presence of oxidants, was used to measure the relative levels of cellular peroxides (Lautraite et al., 2003). NSPCs (1 × 10^5^ cells/mL) were treated with edaravone (0 μM or 100 μM) under hypoxia or without edaravone under normoxia for 24 hr, and then incubated with DCF-DA (10 μM) for 20 min at 37℃ and washed three times with PBS. After centrifugation, the supernatants were removed, and the cells were resuspended with PBS. Flow cytometry (BD Biosciences, San Jose, CA, USA) was used to measure fluorescence at an excitation wavelength of 502 nm and an emission wavelength of 530.

### Western Blot Analysis

To observe HIF-1α and cleaved caspase-3 protein levels, passage 1 neurospheres were incubated with edaravone (0 or 100 μM) under hypoxia or without edaravone under normoxia for 12 hr and 24 hr, respectively. The neurospheres were collected and used for western blot analysis, as described previously (Zheng et al., 2012). The antibodies used included mouse monoclonal anti-β-actin antibody (1:10,000; Sigma-Aldrich, USA), rabbit polyclonal anti-HIF-1α antibody (1:1,000; Abcam, Cambridge, UK), and rabbit polyclonal anti-caspase-3 antibody (cleaved, 1:200; Millipore, USA). The enhanced chemiluminescent substrate (Thermo Scientific Pierce, USA) visualized the immunoreactive bands using horseradish peroxidase-labeled secondary antibodies (1:5,000; Santa Cruz, CA, USA). The housekeeping protein β-actin was used as a control. Luminescent signals detected by the charge-coupled-device camera were transmitted to the controller unit, and the data were sent to the computer for analysis and documentation.

### Quantification and Statistical Analysis

BrdU-positive cells, DCX-positive cells, BrdU/NeuN, BrdU/GFAP, BrdU/TUNEL-positive cells in the SGZ, and histological assessment of both brain hemispheres were counted blindly in five 7 -µm sections per animal, spaced 49 µm apart. Fluorescence images were digitized using laser confocal microscopy (FV1000, Olympus, Japan). The density of positive cells was presented as the total number of positive cells within the SGZ. The purpose of quantification was not to estimate the total number, but to obtain a count within the studied regions to compare between different groups. The measurement data were presented as means ± SD and analyzed by two-tailed Student’s *t*-test or one-way ANOVA, followed by Bonferroni correction. The Mann–Whitney U test analyzed the histological grading scores of severity of neuronal damage. The Kruskal–Wallis test was used for data analysis from cell culture. A *p* value less than .05 indicated a statistically significant difference.

## Results

### Treatment With Edaravone Attenuated CA1 Injury and Improved Neurological Function

**Figure 1.**
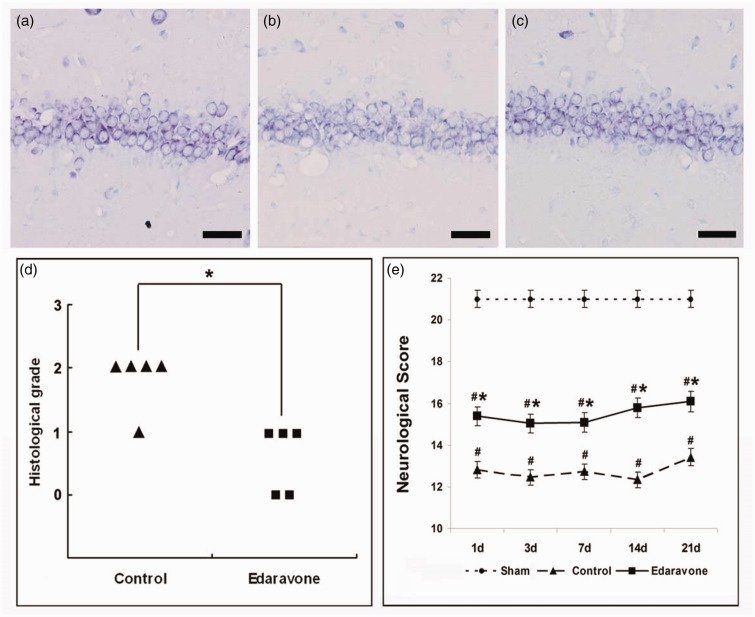
Edaravone attenuated CA1 injury and improved neurological function after global cerebral ischemia. (a–c) Representative photomicrographs of the cresyl violet-stained hippocampal CA1 7 days after ischemia. (a) Sham-operated group. (b) Control group: Prominent neuronal cell loss was observed in the CA1 region. (c) Treatment with edaravone reduced CA1 injury. Scale bar = 25 µm. (d) Histological grading scores 7 days after ischemia, *n* = 5 in each group. (e) Neurological score was tested 1, 3, 7, 14, 21 days after ischemia, *n* = 5. *p < .01 compared with the control group; ^#^*p* < .01 compared with the sham-operated group.

To determine the neuroprotective effect of edaravone against hippocampal neuronal injury, the brains were evaluated histologically 7, 14, and 21 days after ischemia. In the sham-operated group, there was no neuronal loss in the hippocampal CA1 region (Figure 1(a)). However, pronounced neuronal loss was observed in the hippocampal CA1 region of the control group 7 days after ischemia (Figure 1(b)). Treatment with edaravone significantly attenuated CA1 neuronal damage compared with the control group (Figure 1(c) and (d), *p* < .01). There was no significant difference between the control and the edaravone group 14 and 21 days after ischemia (*p* > .05, data not shown). Neurological assessment revealed that all animals displayed the maximum score of 21 points preoperatively. Sham-operated animals were not affected by anesthesia or surgical procedure and retained the maximum score until Day 21 (Figure 1(e)). There was a significant decrease in the neurological score in the ischemic animals from Days 1 to 21, compared with the sham-operated group (Figure 1(e), *p* < .01). The neurological score was significantly higher in the edaravone-treated group at Days 1–21 after ischemia, compared with the control group (Figure 1(e), *p* < .01).

### Treatment With Edaravone Increased Neurogenesis in the SGZ

**Figure 2.**
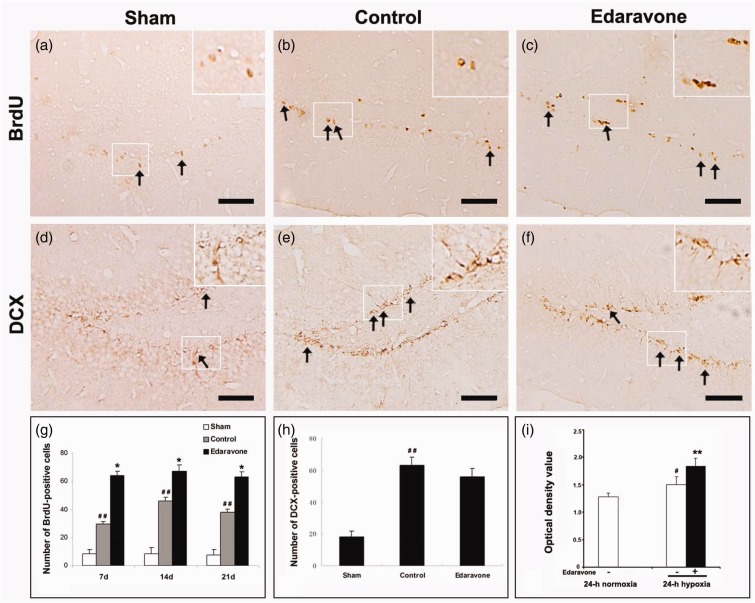
Changes in BrdU- or DCX-positive cells in the SGZ and BrdU incorporation. (a) BrdU-immunoreactive cells in the SGZ of sham-operated group 7 days after sham-operation. (b, c) Representative images of the BrdU-immunoreactive cells in the SGZ 7 days after ischemia*.* BrdU-positive cells show brown granule. (d) DCX-immunoreactive cells in the SGZ of sham-operated group 7 days after sham-operation. (e, f) Representative images of the DCX-immunoreactive cells in the SGZ 7 days after ischemia. (g) Quantification of BrdU-positive cells in the SGZ. (h) Quantification of DCX-positive cells in the SGZ 7 days after ischemia. Arrows indicated positive cells. (i) Quantification of BrdU incorporation by spectrophotometry under different oxygen condition. The experiment was repeated three times. BrdU = 5-bromo-2-deoxyuridine; DCX = doublecortin. Scale bar = 100 µm. * *p* < .05, ** *p* < .01 compared with the control group. *p < .05. **p < .01 compared with the control group. ^#^*p* < .05. ^##^*p* < .01 compared with the sham-operated group or normoxia. *n* = 5 in each group.

**Figure 3.**
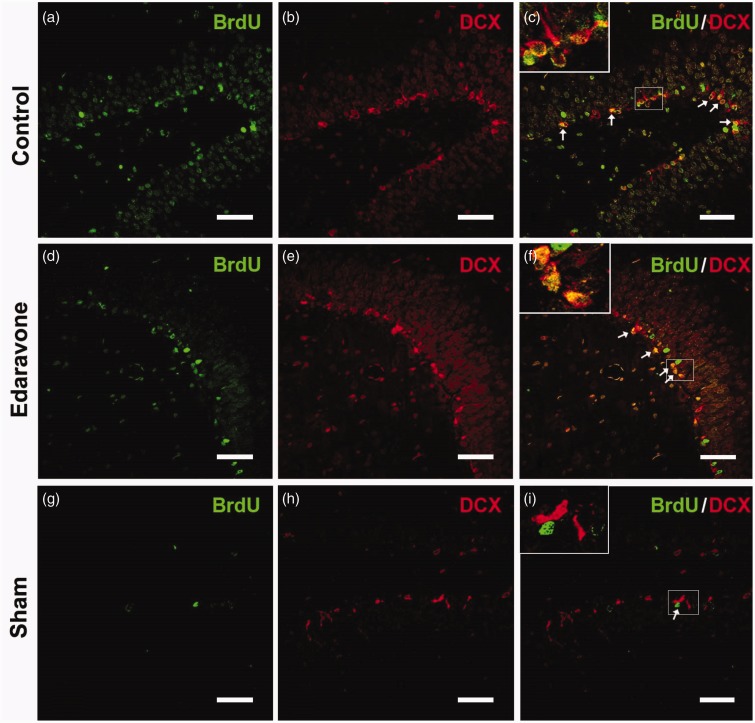
Representative images of BrdU and DCX double immunofluorescence staining 7 days after ischemia. The pictures were from the rats in control group (a–c), edaravone-treated group (d–f), and sham-operated group (g–i). BrdU immunoreactivity (green). DCX immunoreactivity (red). Arrows indicated BrdU/DCX-double-positive cells. BrdU = 5-Bromo-2-deoxyuridine; DCX = doublecortin. Scale bar = 50 µm.

**Figure 4.**
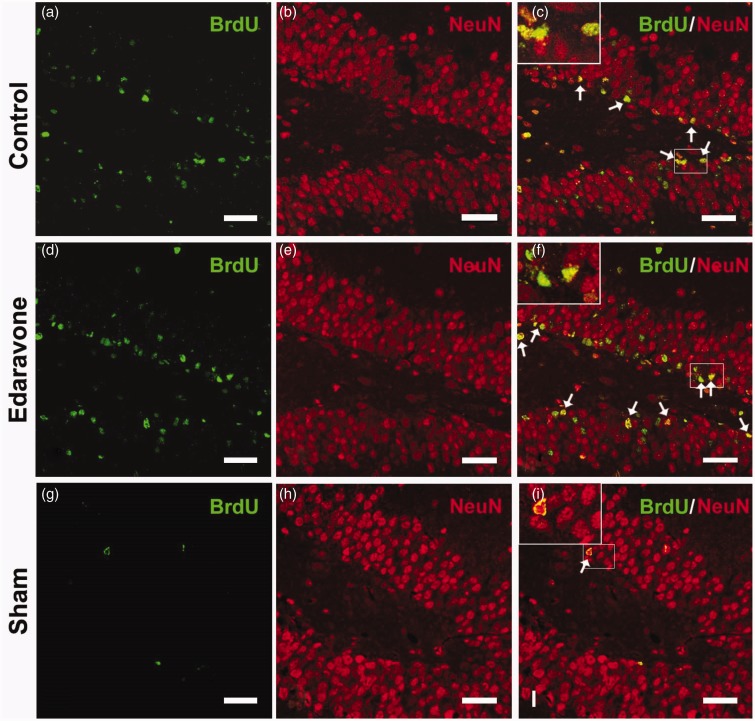
Representative images of of BrdU and NeuN double immunofluorescence staining 7 days after ischemia. The pictures were from the rats in control group (a–c), edaravone-treated group (d–f), and sham-operated group (g–i). BrdU immunoreactivity (green), NeuN immunoreactivity (red). Arrows indicated BrdU/NeuN double-positive cells. BrdU = 5-Bromo-2-deoxyuridine; NeuN = neuron-specific nuclear protein. Scale bar = 50 µm.

**Figure 5.**
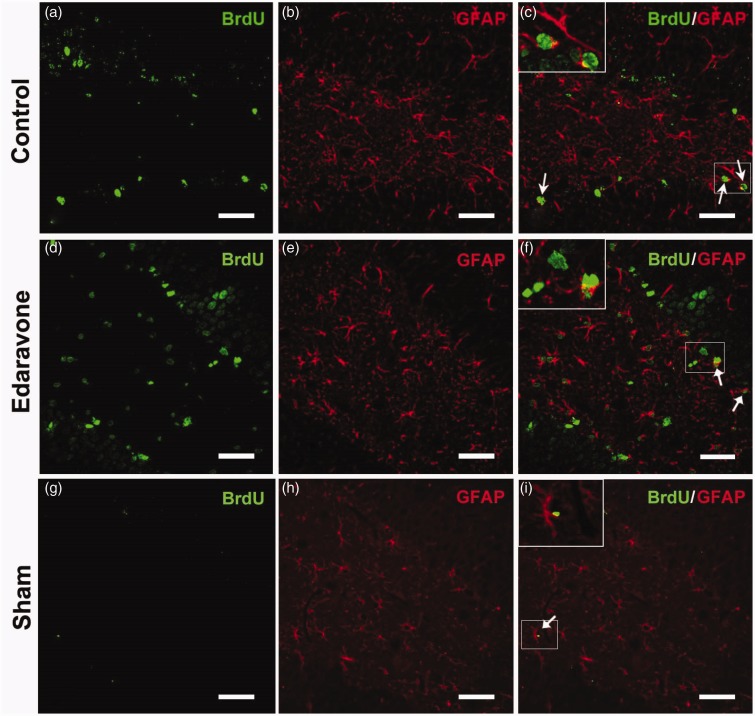
Representative images of BrdU and GFAP double immunofluorescence staining 14 days after ischemia. The pictures were from the rats in control group (a–c), edaravone-treated group (d–f), and sham-operated group (g–i). BrdU immunoreactivity (green). GFAP immunoreactivity (red). Arrows indicated BrdU/GFAP double-positive cells. BrdU = 5-Bromo-2-deoxyuridine; GFAP = glial fibrillary acidic protein. Scale bar = 50 µm.

To detect whether edaravone enhanced neurogenesis in the SGZ, immunohistochemistry was performed at different time points after ischemia. In the sham-operated animals, very few BrdU-labeled cells were found in the SGZ after injection of BrdU for 7 consecutive days (Figure 2(a)). Following ischemia insult, increased BrdU-positive cells were found in the SGZ in both the control and edaravone-treated groups compared with the sham-operated group (Figure 2(b) and (c)). Additionally, edaravone significantly increased the number of BrdU-positive cells in the SGZ compared with the control group during the entire postischemic period (Figure 2(g)). Furthermore, BrdU-positive cells coexpressed DCX (Figure 3(a) to (f)) or NeuN (Figure 4(a) to (f)) in the SGZ, in both the control and edaravone-treated groups 7 days after ischemia. However, very few BrdU-positive cells coexpressing DCX (Figure 3(g) to (i)) or NeuN (Figure 4(g) to (i)) were observed in the SGZ of the sham-operated rats. The number of DCX-positive cells in the SGZ was not significantly different between the control and edaravone-treated groups at 7 days postischemia (Figure 2(h), *p* > .05). Interestingly, the number of BrdU-positive cells expressing NeuN was significantly higher in the SGZ (50.7 ± 3.6 cells/SGZ, *p* < .05) of the edaravone-treated group compared with the control group at 7 days postischemia (28.4 ± 1.0 cells/SGZ). The number of BrdU-positive cells expressing GFAP was not significantly different in the SGZ between the edaravone group (5.8 ± 0.1 cells/SGZ, Figure 5(d) to (f)) and the control group (4.0 ± 0.1 cells/SGZ, Figure 5(a) to (c), *p* > .05) at 14 days after ischemia. There were no BrdU-positive cells coexpressing GFAP in the SGZ of the sham-operated rats (Figure 5(g) to (i)).

### Treatment With Edaravone Reduced Apoptosis of Newly Generated Cells in the Hippocampus

**Figure 6.**
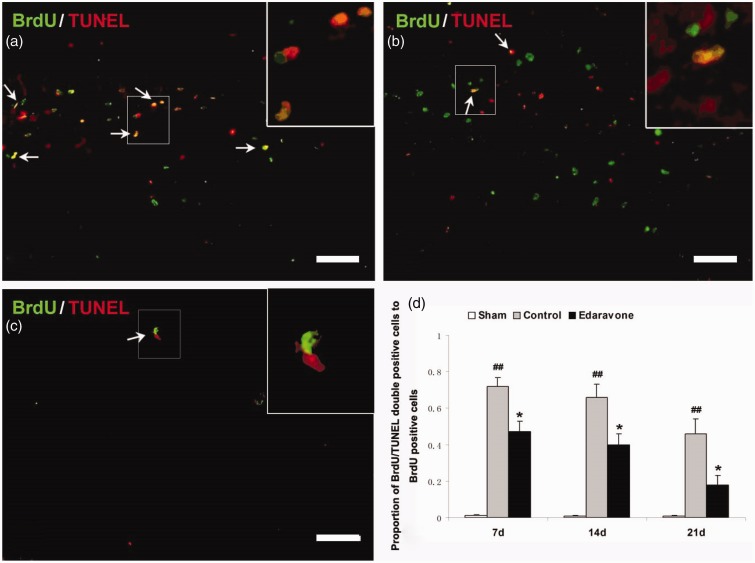
Edaravone reduces apoptosis of newly generated cells in the SGZ. (a–c) Representative confocal images of TUNEL (red) and BrdU (green) double-labeled cells in the control (a), edaravone-treated (b), and sham-operated rats (c). There were nearly no BrdU-TUNEL-positive cells. Fewer of BrdU-TUNEL-positive cells (arrows) were observed in the edaravone-treated rats. Bar = 80 µm. (d) Quantification of BrdU-TUNEL-positive cells in the SGZ of rats 7 days after ischemia. *n* = 5 in each group. *p < .05, compared with the control group, ^##^*p* < .01, compared with the sham-operated group. BrdU = 5-Bromo-2-deoxyuridine; TUNEL = terminal dUTP nick-end labeling.

To determine whether edaravone decreased the apoptosis of newly generated cells, TUNEL and BrdU double staining was performed at defined time points. There were two distinct patterns of TUNEL staining; some TUNEL-stained cells were densely labeled and showed clear apoptotic characteristics. Other TUNEL-stained cells were weakly labeled and were considered necrotic cells. Only densely labeled cells were counted as apoptotic cells (Charriaut-Marlangue and Ben-Ari, 1995). In the sham-operated group, almost no BrdU+/TUNEL+ cells were found in the SGZ (Figure 6(c)). After ischemia, some of BrdU+/TUNEL+ cells were observed in the SGZ of the control (Figure 6(a)) and edaravone-treated groups (Figure 6(b)). The number of BrdU-positive cells expressing TUNEL was significantly lower in the SGZ of the edaravone-treated group than in the control group at all time points examined (Figure 6(d), *p* < .05).

### Characterization of Cultured NSPCs

**Figure 7.**
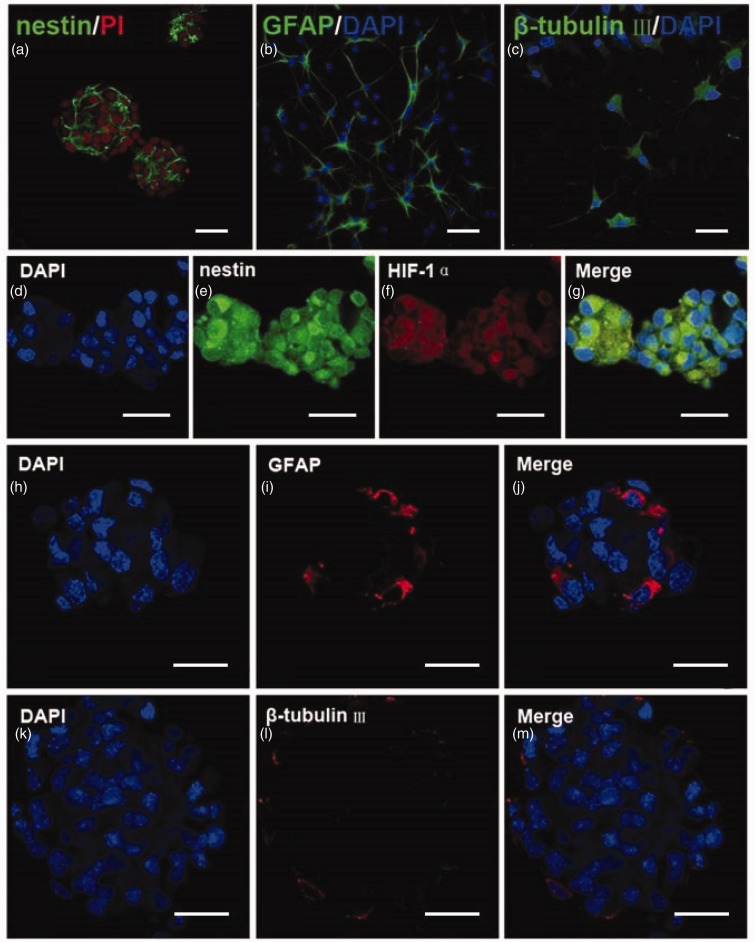
Isolated cells were neural stem cells and passage 1 neurospheres retained the character of NSPCs after hypoxia. (a) Neurospheres formed after 3 days in culture and the vast majority of the neurospheres were nestin-positive (green). (b, c) A subpopulation of NSPCs expressed GFAP (green) or β-tubulin III (green). The nuclei were counterstained with PI (a, red) or DAPI (b, c, blue). Bar = 50 µm in panel (a) and (b). Bar = 30 µm in panel (c). (d–g) Confocal images of the passage 1 neurospheres double-labeled for nestin (green) and HIF-1α (red). The nuclei were counterstained with DAPI (blue). Most of cells in the passage 1 neurospheres were nestin and HIF-1α immunoreactive after 24 hr of hypoxia. (h–j) Confocal microscopic images of the passage 1 neurospheres labeled for marker of astrocytes, GFAP (red). (k–m) Confocal microscopic images of the passage 1 neurospheres labeled for marker of neurons, β-tubulin III (red). The nuclei were counterstained with DAPI (blue). GFAP = glial fibrillary acidic protein; HIF-1α = hypoxia-inducible factor 1α Bar = 50 µm.

Dissociated cells formed neurospheres after 3 days in culture. The vast majority (96 ± 2%) of the passage 1 cells were nestin-positive (Figure 7(a)). After the differentiation assay, a subpopulation of passage 1 cells exhibited immunoreactivity to GFAP (44 ± 5%, Figure 7(b)) and β-tubulin III (21 ± 3%, Figure 7(c)). After placing passage 1 cells in the microaerophilic incubation system for 24 hr, the majority (95 ± 3%) of the passage 1 cells were nestin-positive (Figure 7(d) to (g)). Only a few cells exhibited immunoreactivity to GFAP (3 ± 1%, Figure 7(h) to (j)) or β-tubulin III (2 ± 1%, Figure 7(k) to (m)). Furthermore, these nestin-positive cells were HIF-1α immunoreactive (Figure 7(d) to (g)). These findings suggest that passage 1 cells retain the character of NSPCs after 24 hr of hypoxia. Spectrophotometric measurement of BrdU incorporation *in vitro* showed that BrdU incorporation was increased in hypoxia conditions compared with normoxic condition (Figure 2(i), *p* < .01), indicating that hypoxia stimulated the proliferation of NSPCs.

### Edaravone Decreased NSPC Apoptosis and Declined ROS Generation In Vitro

**Figure 8.**
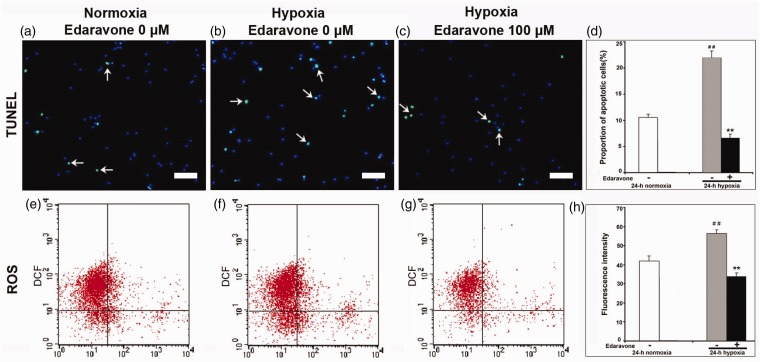
Detection of NSPCs apoptosis and intracellular ROS *in vitro*. (a–c) Images of the cells double-labeled for TUNEL (green) and DAPI (blue). Decreased cell apoptosis was observed in the edaravone-treated cells. Arrows indicated BrdU/TUNEL-double-positive cells. Bar = 50 µm. (d) Quantification of cell apoptosis. (e–g) Endogenous ROS was measured by flow cytometry. (h) Quantification of DCF fluorescence. The experiment was repeated three times. **p < .01 compared with the untreated group. ^##^*p* < .01 compared with normoxia. TUNEL = terminal dUTP nick-end labeling; ROS = reactive oxygen species; DCF = 2,7-dichlorofluorescien.

As shown in Figure 8(a) to (d), hypoxia increased the proportion of apoptotic cells (*p* < .01), and edaravone decreased NSPC apoptosis, as assessed by TUNEL assay (Figure 8(b) to (d), *p* < .01). To estimate the effect of edaravone on ROS generation in NSPCs, passage 1 cells were treated with edaravone (0 μM or 100 μM) under hypoxia or without edaravone under normoxia for 24 hr, and endogenous ROS generation was measured by DCF fluorescence. Hypoxia increased ROS generation compared with normoxia (Figure 8(e), (f), and (h), *p* < .01), and treatment with edaravone led to a significant reduction in DCF fluorescence compared with the control group (Figure 8(f) to (h), *p* < .01). Thus, edaravone significantly reduced intracellular ROS generation after hypoxic insult.

### Edaravone Decreased the Protein Levels of HIF-1α and Cleaved Caspase-3 in NSPCs

**Figure 9.**
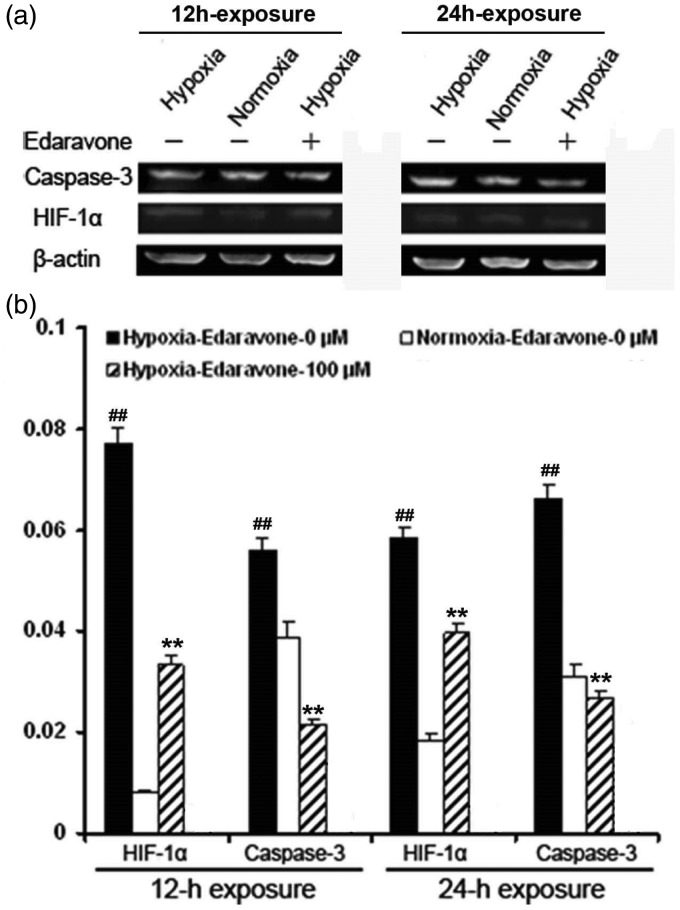
Changes in HIF-1α and caspase-3 protein levels. (a, b) NSPCs were exposed with edaravone (0 μM and 100 μM) for 12 hr or 24 hr under hypoxia or normoxia, respectively. HIF-1α and caspase-3 protein level were analyzed by western blotting. (a) Representative photographs of western blotting. (b) Quantification of HIF-1α and cleaved caspase-3 proteins in NSPCs. The experiment was repeated three times. **p < .01 compared with the untreated group. ^##^*p* < .01 compared with normoxia. HIF-1α = hypoxia-inducible factor 1α.

ROS regulates the HIF-1 signaling pathway, depending on the cell type (Koshikawa et al., 2009). Caspase-3 is one of the genes regulated by HIF-1 and plays an essential role in cell apoptosis (Van Hoecke et al., 2007). To determine whether edaravone decreased HIF-1α and cleaved caspase-3 protein levels, western blot analysis was performed at defined time points. Both HIF-1α and cleaved caspase-3 protein levels significantly increased in NSPCs at 12 hr and 24 hr following hypoxia compared with normoxic conditions, respectively (Figure 9(a) and (b), *p* < .01). NSPCs treated with edaravone showed a significant decrease in HIF-1α and cleaved caspase-3 protein levels compared with untreated cells after hypoxic insult (Figure 9(a) and (b), *p* < .01). These results indicated that edaravone inhibited HIF-1α and cleaved caspase-3 signaling in NSPCs after hypoxia.

## Discussion

In this study, we found that pre- and posttreatment with edaravone attenuated CA1 injury and improved the neurological deficit. Edaravone also increased neurogenesis and reduced apoptosis of newly generated cells in the hippocampus. Furthermore, treatment with edaravone significantly decreased ROS generation, inhibited the protein expression of HIF-1α and cleaved caspase-3, and reduced apoptosis in NSPCs after hypoxic insult. These findings indicated that pre- and posttreatment with edaravone enhances neurogenesis by protecting NSPCs from apoptosis, probably mediated by decreasing ROS generation and inhibiting the protein expression of HIF-1α and cleaved caspase-3 after cerebral ischemia.

The neuroprotective effects of edaravone on cerebral injury have been ascribed to its scavenging of ROS, restoration of the antioxidant defense mechanisms, and antiinflammatory and antiapoptotic effects. Accumulating evidence shows that edaravone ameliorates cortical edema, reduces the infarct volume, and improves neurological deficits in rat focal cerebral ischemia, intracerebral hemorrhage or traumatic brain injury models (Shichinohe et al., 2004; Gao et al., 2009; Itoh et al., 2010). However, the neuroprotective effect of edaravone on global cerebral ischemia has not been assessed. In gerbils subjected to 5 min transient forebrain ischemia, administration of edaravone 30 min before ischemia attenuated neuronal damage and improved behavioral deficits (Lu et al., 2012). By contrast, edaravone administered either immediately or 60 min after ischemia did not change the neurological deficits score in a rat model of global cerebral ischemia induced by 5 min of cardiac arrest and resuscitation (Kubo et al., 2009). Our observation that treatment with edaravone reduced the neuronal damage of CA1 region and improved neurological deficits in rats subjected to 10 min global cerebral ischemia is in agreement with a previous study where edaravone effectively alleviated brain injury and improved the neurological function after focal cerebral ischemia-reperfusion injury (Kikuchi et al., 2009). The discrepancies between outcomes may be related to different global cerebral ischemia models and time of administration used in these studies. Our regimen might be valuable for using edaravone in patients with high risk of predictable global cerebral ischemia, such as cardiac arrest, severe hypotension, or cardiopulmonary bypass.

Protection and amplification of the endogenous neurogenesis expand the possibility of novel neuronal cell regeneration therapies for stroke and other neurological diseases (Bellenchi et al., 2012; Saha et al., 2012). In our study, cerebral ischemia promoted neurogenesis in the SGZ, and edaravone administration increased BrdU-labeled cells and BrdU + /NeuN + cells in the SGZ after 10 min of global cerebral ischemia, indicating that edaravone enhances hippocampal stem/progenitor cell neurogenesis. Interestingly, there was no difference in the number of DCX-positive cells in the SGZ at 7 days postischemia between the control and edaravone-treated groups, indicating that edaravone promoted maturation of new neurons. It was reported that BrdU-positive cells expressing DCX reached their maximum number between the 4th and 7th day after ischemia; thereafter, DCX expression rapidly declined, and then expression of NeuN slowly increased (Brown et al., 2003). Our findings are similar to those of a study showing that edaravone promotes proliferation of neural progenitor cells generated following neuronal loss in the mouse DG (Kikuta et al., 2013) and restores the differentiation of human neural precursor cells following X-irradiation (Ishii et al., 2007). Furthermore, improvement in behavioral deficits was observed in the edaravone-treated rats. However, edaravone administration did not alter gliogenesis at Day 14 postischemia. In contrast to our previous report that treatment with edaravone decreased neurogenesis in the ischemic ipsilateral subventricular zone (SVZ) in a focal cerebral ischemia model (Zhang et al., 2012), the discrepancy can be attributed to the different response of NSPCs in the DG and SVZ to cerebral ischemia/hypoxia. It has been reported that transient global cerebral ischemia significantly increases neurogenesis in the dentate SGZ, but not in the SVZ (Liu et al., 1998). Cell proliferation in the SVZ returned to normal levels; however, that in the SGZ showed a twofold increase 4 weeks following intermittent hypoxia (Zhu et al., 2005). Taken together, the results suggest that neuroprotection of NSPCs by edaravone in the DG and SVZ is different.

Global cerebral ischemia resulted in a decrease of oxygen tension in the hippocampus (Freund et al., 1989). Following reperfusion, oxygen tension recovered within 1 hr in the hippocampus and fell again, often below the lowest level seen during the ischemic period, which initiated histological damage observed after 24 hr (Freund et al., 1989; Dijkhuizen et al., 1998). Both hypoxia and free radicals induce apoptosis in cultured NSPCs (Tamm et al., 2008; Chen et al., 2010). We observed that treatment with edaravone markedly decreased NSPC apoptosis in the hippocampus after ischemia, which agreed with a report that edaravone protected neural progenitor cells in the SGZ of the hippocampus from cell death after X-irradiation (Motomura et al., 2010). Additionally, we simulated the oxygen levels in the hippocampus following global cerebral ischemia/reperfusion to investigate the effect of edaravone on NSPC apoptosis and its potential mechanism *in vitro* (Chen et al., 2010; Tian et al., 2010; Zhao et al., 2012). Hypoxia stimulated proliferation of NSPCs, and treatment with edaravone increased hypoxia-induced NSPC proliferation (data not shown) and decreased cell apoptosis and ROS generation, indicating that enhanced SGZ neurogenesis by edaravone treatment was partly attributed to reduced cell apoptosis. It was reported that ROS induced HIF-1α expression through PI3K-PKB/Ak signaling (Charriaut-Marlangue and Ben-Ari, 1995). HIF-1α is involved in ischemia/hypoxia-induced cell death events by activating the expression of various pro-death genes during sustained or severe ischemia/hypoxia (Wang et al., 2004). A previous study suggested that there was a causal relationship between HIF-1α and caspase-3 induction via HIF-1α functional binding to the caspase-3 gene promoter (Van Hoecke et al., 2007). In our study, treatment with edaravone led to a decrease in intracellular ROS generation, decreased protein levels of HIF-1α and cleaved caspase-3 and reduced NSPC apoptosis under hypoxia, indicating that administration of edaravone protect NSPCs from apoptosis, probably by inhibiting HIF-1α and cleaved caspase-3 pathway.

In conclusion, using a model of transient cerebral ischemia and hypoxic culture of NSPCs, we found that pre- and postadministration of edaravone mitigated hippocampal CA1 injury and enhanced neurogenesis by protecting NSPCs from apoptosis, which was probably mediated by decreasing ROS generation and inhibiting protein expressions of HIF-1α and cleaved caspase-3 after cerebral ischemia. Our results suggest a novel ROS-dependent HIF-1α-mediated caspase-3 activation pathway that leads to NSPC apoptosis and provides an experimental basis for the use of edaravone as a preventive and protective drug against global cerebral ischemia.
